# A Case of Babesiosis in a Pregnant Patient Treated with Red Blood Cell Exchange Transfusion

**DOI:** 10.1155/2019/9869323

**Published:** 2019-05-30

**Authors:** Baruch Abittan, Aaron Nizam, Michael Oey, Felicia Callan, Lisa Simmonds, Sarah L. Pachtman

**Affiliations:** ^1^Donald and Barbara Zucker School of Medicine at Hofstra/Northwell, Department of Obstetrics and Gynecology, North Shore University Hospital, Manhasset, NY, USA; ^2^Donald and Barbara Zucker School of Medicine at Hofstra/Northwell, Department of Internal Medicine, Division of Infectious Diseases, North Shore University Hospital, Manhasset, NY, USA; ^3^Donald and Barbara Zucker School of Medicine at Hofstra/Northwell, Department of Obstetrics and Gynecology, Huntington Hospital, Huntington, NY, USA

## Abstract

Babesiosis, caused predominantly by* Babesia microti*, is an emerging health risk in the Northeastern and Midwestern United States. We present a case of a pregnant woman with history of splenectomy diagnosed with babesiosis at 23 5/7 weeks of gestational age refractory to antimicrobial therapy. She underwent the first reported red blood cell exchange transfusion for babesiosis in pregnancy, at 24 4/7 weeks of gestational age, which resulted in resolution of parasitemia. She had a full term, uncomplicated cesarean delivery. Exchange transfusion is potentially a safe treatment option for severe babesiosis infection in pregnancy and should be considered when other methods are poorly tolerated or ineffective.

## 1. Introduction

Babesiosis is a protozoal infection caused predominantly by* Babesia microti* in North America. Transmitted by the* Ixodes scapularis* tick, babesiosis is endemic in the Northeastern and Midwestern United States. Clinical presentation ranges from mild to severe and occasionally fatal; severity of infection usually depends on the host's immune status [1]. Healthy adults affected by babesiosis may be asymptomatic or develop mild malaise, fatigue, and fevers. Immunocompromised, elderly, or asplenic patients may develop severe disease causing hematologic, pulmonary, renal, or hepatic impairment [2]. Few cases of acute babesiosis in pregnancy are documented and there is no consensus for the optimal treatment or duration of therapy. We present a case of a surgically asplenic patient at 23 5/7 weeks of gestational age diagnosed with acute babesiosis after her partner reported a possible tick bite on Long Island, New York, requiring intensive care admission for red blood cell exchange transfusion after infection was nonresponsive to standard antimicrobial therapy.

## 2. Case

A 32-year-old Caucasian female, G3 P1011 with a singleton intrauterine pregnancy at 23 5/7 weeks of gestation presented with acute onset of fever to 39.5 degrees Celsius, fatigue, and frontal headache during the summer. Remote medical history was significant for a benign pancreatic mass abutting the spleen that was treated surgically with removal of mass along with partial pancreatectomy and total splenectomy. She lived with her partner in a wooded area of New York State and reported daily trips to the beach. She denied any rashes, insect, or tick bites, but her husband recalled seeing a tick on his leg a few days prior to her presentation. She denied rash, myalgias, arthralgia, gastrointestinal symptoms or anorexia, sore throat, dry cough, neck stiffness, abdominal pain, dark urine, conjunctival injection, shortness of breath, or hyperesthesia. Prenatal care had been otherwise uncomplicated.

On initial laboratory evaluation, white blood cell count was 7.9 [K/uL], hemoglobin 12.5 [mg/dL], platelet count was 219 [K/uL], alanine aminotransferase (ALT) 77 [U/L], and aspartate aminotransferase (AST) 84 [U/L]. Peripheral blood smear revealed red blood cell intracellular parasites morphologically consistent with* babesia *species. Laboratory test results were consistent with likely acute infection by* babesia *serologically, with positive tests for IgM antibodies and IgG antibodies (each at a titer of > 1:1024 by immunofluorescence), and real-time polymerase chain reaction (PCR) was positive for* Babesia microti* (Mayo Clinic Laboratories, Rochester, MN). The DNA target for the PCR assay used by this laboratory is a gene encoding the nuclear small subunit ribosomal RNA (SS-rDNA) specific to* babesia* species. Initial parasite density was 1%, consistent with mild disease. Serum serology was negative for* B. burgdorferi *(Lyme disease), and Lyme IgG/IgM antibody index was 0.08 [0.01-0.89 index]. Maternal serum IgG/IgM antibodies for both* Anaplasma phagocytophilum *and* Ehrlichia chaffeensis* were <1:64 [reference <1:64 titers] and <1:20 [reference <1:20 titers], respectively, (Mayo Clinic Laboratories, Rochester, MN). Treatment was initiated with oral atovaquone 750 mg twice a day and oral azithromycin 500 mg once, followed by 250 mg daily. Fetal assessment was normal and weight was appropriate for gestational age. Umbilical artery and middle cerebral artery Doppler studies were normal (S/D 2.3 and MCA 1.38 MoM, respectively).

Parasite density was monitored daily and initially increased to 6% over the first three days of therapy. Antibiotic regimen was transitioned to quinine 648 mg every eight hours and oral clindamycin 600 mg every eight hours because of concern for vertical transmission with subsequent fetal sequelae and rapidly increasing maternal parasitemia despite therapy. Electrocardiogram was performed daily, as quinine is known to prolong the QT interval at standard doses [3]. After six days of antimicrobial therapy, she continued to experience fevers and rigors. Hemoglobin decreased to 8.9 [mg/dL], lactate dehydrogenase [LDH] >2000 [U/L], and serum haptoglobin < 20 [mg/dL]. The platelet count was mildly depressed but did not decrease below 125 [K/uL] at any point during her hospital course. Total bilirubin level was also elevated to 2.8 [mg/dL], consistent with hemolytic anemia. On the seventh day of therapy, oral atovaquone 750 mg twice a day was added to the quinine and oral clindamycin regimen in attempt to treat persistently rising parasitemia and worsening hemolysis.

Total red blood cell exchange procedure was performed because infection was refractory to standard antibiotic therapy for this extended time period. Central venous access was obtained and red blood cell exchange was performed at 24 4/7 weeks of gestation with six units of packet red blood cells. Fetal status was monitored and was reassuring before and after exchange transfusion.

Following red blood cell exchange transfusion, daily maternal serum parasite density measured <1% for four consecutive days. Oral azithromycin was substituted for quinine because of maternal QT prolongation (corrected QT interval 647 [ms]) and concern for quinine-induced hemolysis. Maternal QT interval returned to baseline within 24 hours of discontinuation of quinine. After improvement in her clinical status she was discharged from the hospital with oral atovaquone 750 mg two times a day, oral azithromycin 250 mg daily, and oral clindamycin 600 mg every eight hours. Her treatment course is outlined in Table 1. Following therapy, she continued to be anemic secondary to continued hemolysis despite resolution of parasitemia with hemoglobin 7.4 [mg/dL], reticulocytes 7.2 [%], total bilirubin 4.4 [mg/dL], lactate dehydrogenase 2369 [U/L], and serum haptoglobin <20 [mg/dL] and so received simple transfusion of 2 units of packed red blood cells. Maternal blood type was Rh negative, and Rh immune globulin was administered at 32 weeks of gestational age instead of our standard 28 weeks of gestational age because of transfusions. Following six weeks of antimicrobial therapy she was transitioned to daily oral azithromycin 250 mg for the duration of her pregnancy, with all subsequent peripheral blood smears negative for parasites.

Labor was induced electively with prostaglandins and oxytocin at 40 weeks of gestational age. Primary cesarean delivery for nonreassuring fetal status was uncomplicated. Postpartum repeat PCR for* Babesia *species (Mayo Clinic Laboratories, Rochester, MN) and peripheral blood smear were negative and so azithromycin was discontinued. Placental pathology noted a heavy for gestational age placenta and was significant for many mononuclear cells with folded nuclei in fetal vasculature with occasional nucleated erythrocytes, low grade chronic villitis with lymphohistiocytic inflammation, and chorioamniotic membranes with scattered macrophages. Immunohistochemical staining of mononuclear cells suggested a mixture of myeloid, monocytic, and erythroid cell lines consisted with a leukoerythroblastic reaction.

At time of delivery, the neonate was noted to have a diffuse, generalized nodular “blueberry muffin” petechial rash on his body including face, palms and soles, trunk, and all four extremities. Hepatomegaly was also noted on physical exam. Laboratory evaluation was notable for thrombocytopenia of 79 [K/uL], elevated lactate dehydrogenase to 3650 [U/L] and reticulocyte percent 6.3 [%], and elevated AST of 152 [U/L].

Initially thought to be secondary to vertical transmission of babesiosis, the neonate was treated with antibiotic therapy for three days until testing (including peripheral blood smears for parasites, real-time* Babesia* species PCR, and* Babesia* IgM antibodies [reference <1:20]) resulted negative (Mayo Clinic Laboratories, Rochester, MN). Neonatal* Babesia* IgG antibody was 1:256 [reference <1:64] which was attributed to passive placental passage. Other possible causes of “blueberry muffin” rash were also considered and evaluation for other infectious etiologies such as herpes simplex virus, rubella, parvovirus, cytomegalovirus and Lyme, Ehrlichia and Anaplasmosis, and noninfectious etiologies were negative. Skin biopsy was performed that confirmed extramedullary hematopoiesis. Etiology was attributed to Rh-incompatibility given maternal Rh negative and fetal Rh positive and Coomb's positive status. The neonate was discharged after monitoring in the neonatal intensive care unit and had close follow-up with pediatric infectious disease, dermatology, and hematology services with improvement of petechial rash at time of discharge.

## 3. Discussion

This case outlines the challenge of successfully treating an asplenic, immunocompromised patient with confirmed babesiosis during pregnancy and demonstrates that red blood cell exchange transfusion can be effective. Severe sequelae including hemolytic anemia can occur in patients infected with* Babesia microti* after splenectomy [2, 3]. Laboratory values in this case revealed a normal white blood cell count and elevated transaminase levels, consistent with previously reported findings [4]. Quinine and clindamycin combination therapy is the most widely accepted treatment of babesiosis in pregnancy because of improved placental penetration and the potential capability to prevent vertical transmission [5]. The incidence of QT prolongation and Torsade de Pointes with quinine use is largely unknown due to underreporting [6, 7]. In this case, the patient was closely observed on continuous electrocardiogram monitoring and had significant QT prolongation which required discontinuation of quinine.

Medical treatment was transitioned first with the addition of oral atovaquone and then to oral atovaquone, oral azithromycin, and oral clindamycin because of inadequate response to initial therapy with quinine and clindamycin. In nonpregnant patients presenting with mild disease, treatment with atovaquone and azithromycin is the preferred treatment [4, 8] with good reported outcomes and so this treatment strategy was employed. After these regimens proved to be ineffective, red blood cell exchange transfusion was attempted as a treatment effort because of reported efficacy in the nonpregnant population.

Red blood cell exchange transfusion has been used as therapy in nonpregnant patients with babesiosis and severe hemolysis, parasitemia greater than ten percent, or hepatic, pulmonary, or renal impairment [9–13]. There are few case series studying exchange transfusion in nonpregnant patients with babesiosis, and we believe this is the first reported case of exchange transfusion to treat babesiosis during pregnancy. Exchange transfusions can reduce parasitic load, correct anemia, and remove toxic products secondary to red blood cell lysis [10, 14]. Exchange transfusion during pregnancy has been reported twice as successful treatment of severe falciparum malaria [15, 16] and has also been described for severe sickle cell crisis during pregnancy [17] with good outcomes. Decision was made to utilize red blood cell exchange transfusion in this case due to failure of antibiotic therapy and increasing parasitemia, persistent fevers, and worsening hemolytic anemia. In this case, the patient was monitored in an intensive care setting for the duration of the exchange transfusion. After red blood cell exchange, parasite count decreased significantly and hemolysis slowly resolved and the patient eventually had a complete response to this treatment.

Babesiosis in pregnancy, especially in the setting of maternal splenectomy, can be a devastating disease for both the mother and developing fetus that is difficult to treat. Antibiotic therapy can be effective treatment and potentially reduce the risk of vertical transmission, however, occasionally requiring a prolonged course and therefore subjecting both mother and fetus to consequences of infection. We believe that this is the only report of exchange transfusion to treat this type of protozoal infection in a pregnant patient and provides an example of its successful use. Exchange transfusion is potentially a safe and effective treatment option for severe babesiosis infection in pregnancy and can be considered when other methods are poorly tolerated or ineffective.

## Figures and Tables

**Table 1:** Percentage of *Babesia* parasitemia during the course of treatment.

	Treatment	Parasitemia (%)
Hospital Day 1	Initial peripheral blood smear performed Atovaquone + azithromycin	1%

Hospital Day 3	Quinine + clindamycin	6%

Hospital Day 7	Quinine + clindamycin + atovaquone Total red blood cell exchange transfusion	3%

Hospital Day 8	Azithromycin + clindamycin + atovaquone	<1%

Hospital Day 9	Azithromycin + clindamycin + atovaquone	<1%

Hospital Day 12	Azithromycin + clindamycin + atovaquone Simple red blood cell transfusion	<1%

Hospital Day 14	Azithromycin + clindamycin + atovaquone	Undetectable
